# Syntaxin1A overexpression and pain insensitivity in individuals with 7q11.23 duplication syndrome

**DOI:** 10.1172/jci.insight.176147

**Published:** 2024-02-22

**Authors:** Michael J. Iadarola, Matthew R. Sapio, Amelia J. Loydpierson, Carolyn B. Mervis, Jill C. Fehrenbacher, Michael R. Vasko, Dragan Maric, Daniel P. Eisenberg, Tiffany A. Nash, J. Shane Kippenhan, Madeline H. Garvey, Andrew J. Mannes, Michael D. Gregory, Karen F. Berman

**Affiliations:** 1 Department of Perioperative Medicine, Clinical Center, National Institutes of Health (NIH), Bethesda, Maryland, USA.; 2 Neurodevelopmental Sciences Laboratory, Department of Psychological and Brain Sciences, University of Louisville, Louisville, Kentucky, USA.; 3 Department of Pharmacology & Toxicology, Indiana University School of Medicine, Indianapolis, Indiana, USA.; 4 Flow and Imaging Cytometry Core Facility, National Institute of Neurological Disorders and Stroke (NINDS), and; 5 Clinical and Translational Neuroscience Branch, National Institute of Mental Health (NIMH), NIH, Bethesda, Maryland, USA.

**Keywords:** Genetics, Neuroscience, Molecular genetics, Pain, Synapses

## Abstract

Genetic modifications leading to pain insensitivity phenotypes, while rare, provide invaluable insights into the molecular biology of pain and reveal targets for analgesic drugs. Pain insensitivity typically results from Mendelian loss-of-function mutations in genes expressed in nociceptive (pain-sensing) dorsal root ganglion (DRG) neurons that connect the body to the spinal cord. We document a pain insensitivity mechanism arising from gene overexpression in individuals with the rare 7q11.23 duplication syndrome (Dup7), who have 3 copies of the approximately 1.5-megabase Williams syndrome (WS) critical region. Based on parental accounts and pain ratings, people with Dup7, mainly children in this study, are pain insensitive following serious injury to skin, bones, teeth, or viscera. In contrast, diploid siblings (2 copies of the WS critical region) and individuals with WS (1 copy) show standard reactions to painful events. A converging series of human assessments and cross-species cell biological and transcriptomic studies identified 1 likely candidate in the WS critical region, *STX1A*, as underlying the pain insensitivity phenotype. *STX1A* codes for the synaptic vesicle fusion protein syntaxin1A. Excess syntaxin1A was demonstrated to compromise neuropeptide exocytosis from nociceptive DRG neurons. Taken together, these data indicate a mechanism for producing “genetic analgesia” in Dup7 and offer previously untargeted routes to pain control.

## Abstract 2

<p>Overexpression of syntaxin1A in individuals with 7q11.23 duplication syndrome&nbsp;produces insensitivity to a broad spectrum of painful injuries.</p>

## Graphical abstract

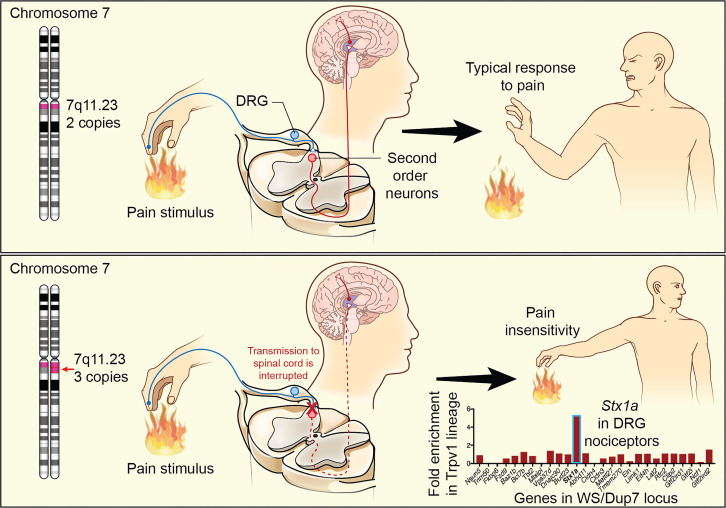

## Introduction

Pain is the perception and processing of noxious physical stimuli, such as hot, cold, mechanical, chemical, inflammatory, and tissue-damaging insults. These algesic stimuli, encountered externally in the environment and internally because of disease, tissue damage, or inflammation (1), are transduced by an array of ligand- and voltage-gated ion channels, and chemoreceptors in specialized peripheral sensory neurons, termed nociceptors. These sensory afferents have their cell bodies in the dorsal root ganglia (DRGs) and synapse in dorsal spinal cord. Collectively these elements are referred to as the nociceptive system (2–5). Several monogenic loss-of-function mutations in genes expressed in DRG sensory neurons are known to reduce or eliminate nociceptive sensitivity by interrupting transmission at this crucial first step in the pathway to perception, providing critical genetic insight into the molecular biology of nociception (5–7). Mechanistic insights gleaned from several of these genetic mutations (e.g., sodium channels and nerve growth factor receptor) have provided genetically verified leads for development of peripherally acting analgesic agents (8–10) that are designed to avoid CNS side effects.

In contrast with identified loss-of-function mutations, evidence for a genetic overexpression mechanism that confers pain insensitivity has yet to be identified. The central hypothesis of this study is that interference with pain detection in individuals with 7q11.23 duplication syndrome (Dup7) stems, at least in part, from genetically driven overexpression of a gene in the duplicated segment in DRG neurons, thereby preventing entry of nociceptive signals into the spinal cord, resulting in analgesia. We demonstrate that people with Dup7, who have 3 copies (+/+/+) of the 25 to 27 genes in the 7q11.23 Williams syndrome (WS) locus (11–13), consistently show clinically meaningful insensitivity to painful stimuli and tissue injury events. Dup7 and WS are rare copy number variants (CNVs) arising from genetic duplication or hemideletion, respectively, of the genes in the CNV due to low copy number repeats flanking both ends of the locus, which predispose to homologous recombination errors during meiosis (13). Phenotypic features of individuals with WS (1 copy of these genes) include high social drive, supravalvar aortic stenosis (due to hemizygous loss of elastin), and characteristic facial features (13, 14). In contrast, individuals with Dup7 (3 copies of the genes) present with an asocial profile, aortic aneurysms or dilation, and different but distinct facial features, among other characteristics (15). The goals of the present research were to identify which of the genes in the 7q11.23 CNV might account for the nociceptive deficiency seen in the individuals with Dup7 and to explore molecular mechanisms.

To address these questions, DRG neurons were divided into nociceptive and non-nociceptive subpopulations and transcriptomically profiled for expression of gene(s) in the 7q11.23. Only 1 transcript, Syntaxin 1A (*STX1A*), showed clear enrichment in the nociceptive neuronal population. This stimulated the hypothesis that pain insensitivity in people with Dup7 is, at least in part, due to overexpression of *STX1A* in DRG nociceptors. The encoded protein syntaxin1A is a particularly compelling candidate because, as part of the soluble *N*-ethylmaleimide–sensitive fusion protein (NSF) attachment protein receptor (SNARE) complex, it is a critical molecular component for fusion of large, dense-core synaptic vesicles with the presynaptic membrane. Vesicle docking and fusion are necessary steps for release of transmitters and neuropeptides from primary afferent nerve terminals. This multicomponent molecular event is carefully orchestrated (16–18), and viral overexpression of *STX1A* was used to test the hypothesis that increased syntaxin protein levels can inhibit noxious stimulus–evoked synaptic release of neuropeptides from nociceptive primary afferent neurons. The results support the idea that inhibition of transmitter release from DRG presynaptic afferent synapses in dorsal spinal cord is the basis for the genetic overexpression analgesia observed in individuals with Dup7.

## Results

### Painless injuries in people with Dup7 and parental descriptions of painlessness.

Twenty-four individuals with rare 7q11.23 CNVs — 11 with Dup7 (+/+/+) and 13 with WS (+/–), as well as 16 unaffected, typically developing siblings — were assessed to gauge injury-related responses. All but 2 of the 40 participants were children (Table 1). In view of their young age, participants were not evaluated using experimental pain tests. Two of the 8 child participants with Dup7 had been referred for genetic testing for pain insensitivity syndromes based on their nonreaction to an injury that should have resulted in extreme pain. The resulting microarray test disclosed the presence of the Dup7 CNV.

**Figure 1:**
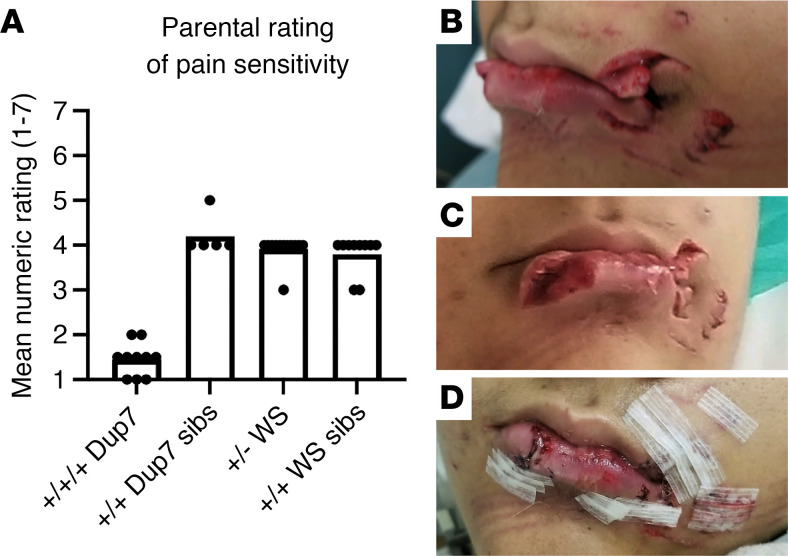
Ratings of insensitivity in individuals with WS or 7q11.23 duplication or their siblings. (**A**) Average and individual data p oints of parental rating of pain response following tissue-damaging accidents or other pain-producing events. Significantly lower pain sensitivity is reported for individuals with 3 copies of the 7q11.23 locus (*P* < 0.0001, Kruskal-Wallis; +/+/+ Dup7, *n* = 10; +/+ Dup7 sibs, *n* = 5; +/– WS, *n* = 12; +/+ WS sibs, *n* = 10). sibs, siblings. (**B**–**D**) Example of tissue damage from dog bites that did not produce a pain reaction in this individual with Dup7 despite copious bleeding and the necessity for surgical repair (**D**).

The 2 adults with Dup7 and the parents of the children with Dup7 were asked to rate their perception of the degree of pain sensitivity using a 7-point scale developed for this study. A rating of 1 indicated “seems extremely insensitive to pain,” a 4 “seems to feel pain at the level expected for the situation and the participant’s age,” and a 7 “seems extremely sensitive to pain” (Figure 1A). The mean ratings were 1.41 ± 0.11 (range: 1–2) for individuals with Dup7, 3.92 ± 0.08 (range: 3–5) for children with WS, and 3.94 ± 0.11 (range: 3–4) for typically developing siblings (*P* < 0.0001, Kruskal-Wallis; Figure 1A). Alongside this assessment, parents also provided narrative descriptions of painless injuries. The number of reported painless instances described for each group was 67 reports for individuals with Dup7 and none for individuals with WS or for the siblings of either group, except for 1 report in a sibling of a child with WS (Table 1).

In individuals with Dup7, precipitating conditions, accidents or injuries were often severe, and the lack of observable response was clearly alarming to parents, partners, and treating physicians. Excerpts from parental reports included the following: a) A child was severely bitten by a dog on the face yet exhibited no painful reaction. Photographic documentation of this injury is provided in Figure 1, B–D, with permission. b) “He hit his jaw and knocked a tooth out. He was not fazed at all – just showed [his mother] the tooth. Blood was pouring out of his mouth… he complained about the taste of the blood.” c) “We suddenly ended up in the emergency room (for severe constipation) while the family was traveling.” Mother noticed that X was pale and felt cold to the touch. “Anyone else would have complained of pain for days before. The emergency room doctor was very surprised that X had not complained; he said the pain should have been severe.” d) “Almost completely insensitive to pain.” e) A child got into the bathtub before his mother had a chance to check the water temperature. When she got into the bathroom, he was red. She made him get out. All he said was, “Look, Mom. I’m red.” f) A mother noticed that her child was walking “funny.” She looked down at his feet and saw that one was at an odd angle. “He never complained.” A subsequent x-ray indicated his ankle was broken.

The diversity of organ systems impacted demonstrates that nociceptive insensitivity in people with Dup7 is a body-wide phenomenon. Moreover, the insensitivity affects multiple nociceptive modalities, including heat pain, soft tissue damage, accidents to bone or teeth, and visceral pain. Thus, a sufficiently explanatory operative mechanism must account for the distributed nature and multimodality (e.g., heat, visceral, mechanical injury) of the insensitivity phenotype.

### Expression of 7q11.23 WS/Dup7 locus genes in DRG identifies STX1A as a candidate target.

**Figure 2:**
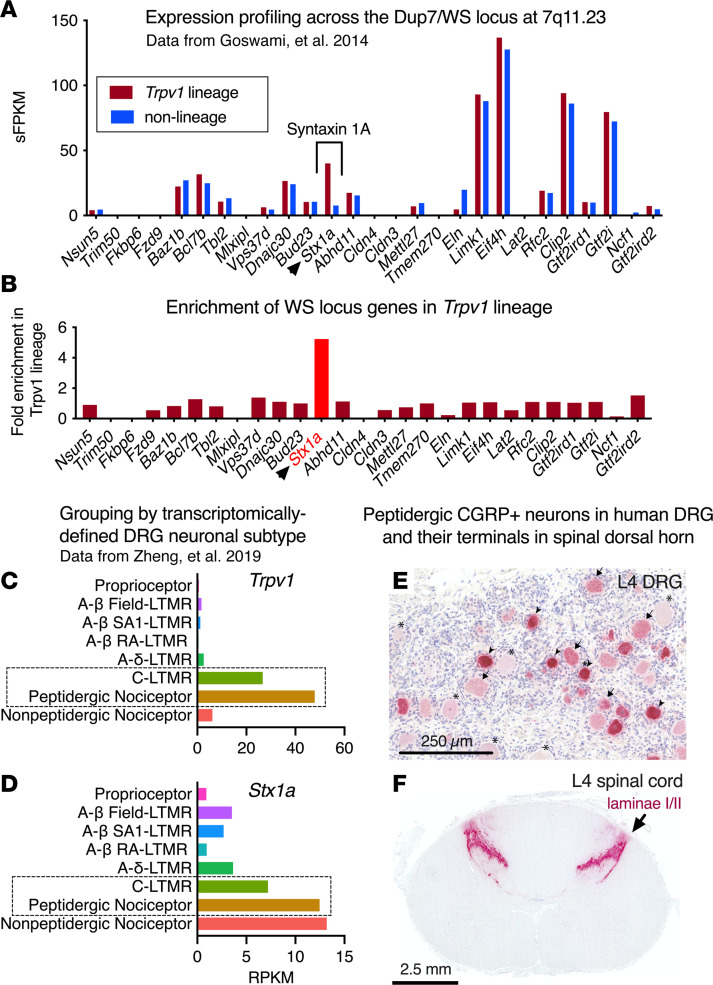
Transcriptomic expression profile of WS/Dup7 genes in DRG and peptidergic innervation of human spinal cord. (**A** and **B**) Expression across the 7q11.23 locus in genetically fluorescent mouse DRG neurons separated by FACS into *Trpv1*^+^ lineage and non-*Trpv1*^+^ cells. (**A**) *Stx1a* was the only gene in 7q11.23 exhibiting enriched expression in the *Trpv1* neuronal lineage whereas the others were either equally or not expressed in the 2 populations. (**B**) An approximately 6-fold enrichment is evident for *Stx1a* compared with other genes in the locus. (**C** and **D**) Bioinformatic analyses of a second genetically labeled mouse DRG neuron study. Labeled neurons were separated into 8 phenotypic groups (24). The groups expressing *Trpv1* or *Stx1a*, respectively, are shown in **C** and **D**. *Stx1a* is detected in the 2 main subgroups that express *Trpv1* (dotted boxes, **C** and **D**) and in a nonpeptidergic group of nociceptors. Abbreviations in **C** and **D** relate to sensory modality and axon type. A-β Field LTMR, large-fiber, low-threshold mechanoreceptor responsive to skin stroking; A-β SA1-LTMR, slowly adapting LTMR; A-β RA1-LTMR, rapidly adapting LTMR; A-δ LTMR, lightly myelinated LTMR; C-LTMR, small, unmyelinated C-fiber LTMR; peptidergic nociceptor, peptide-containing nociceptor (generally C-fiber); nonpeptidergic nociceptor: pain-sensing C-fiber neurons without peptides (71, 72). (**E**) CGRP staining of human DRG neurons overlaps extensively with TRPV1 and serves as a surrogate marker for TRPV1^+^ neurons. A range of staining from lightly labeled (arrowheads) to darkly labeled (arrows) and unlabeled neurons (asterisks), and a range of sizes, are seen. This is similar to the pattern of staining for *TRPV1* in the human as seen in Figures 6 and 7 using in situ hybridization. (**F**) The axonal projections of CGRP-expressing DRG neurons densely terminate in the superficial layers of human spinal cord dorsal horn, the spinal region that processes nociceptive peripheral input. sFPKM, significant fragments per kilobase per million aligned reads; RPKM, reads per kilobase of transcript per million bases sequenced.

Given our working hypothesis that the DRG is the most plausible site for genetic causes of painlessness, we used transcriptomic expression profiling to ascertain which gene(s) in the 7q11.23 WS/Dup7 locus exhibit enriched expression in the nociceptive population of DRG neurons. We focused on transient receptor potential vanilloid 1–expressing (*TRPV1*-expressing) neurons (Figure 2, A–D) because they are known to transduce thermal heat, inflammatory stimuli, pain from advanced cancer, osteoarthritis, and surgical incision (19–21), a range that overlaps the nociceptive insensitivity in individuals with Dup7. Furthermore, pharmacological chemo-inactivation of TRPV1^+^ neurons (1, 22) produces robust analgesia in multiple pain indications in animals and humans, paralleling the broad inhibition of nociception seen in individuals with Dup7.

We therefore examined which gene(s) in the 7q11.23 WS/Dup7 locus was differentially expressed in TRPV1^+^ nociceptive neurons by analyzing murine DRG neuronal preparations that were sorted into nociceptive and non-nociceptive populations. Genetically tagged neurons were separated into a nociceptive *Trpv1*^+^ neuronal lineage and a second population containing *non*-*Trpv1*-expressing proprioceptive neurons, satellite glial cells, and myelin-producing Schwann cells. Each of the 2 populations was analyzed by deep RNA sequencing (23). Figure 2, A and B, shows that only *Stx1a* exhibited enriched expression (6-fold) in the *Trpv1*^+^ neuronal lineage compared with non-nociceptive cells (Figure 2B). The remaining 26 genes in the WS/Dup7 locus were not expressed in DRG or were evenly expressed in the 2 populations. This result was verified using a second data set (24) generated from RNA sequencing of 8 groups of genetically labeled, physiologically categorized, and sorted DRG neurons (Figure 2, C and D). Here, too, *Stx1a* expression was enriched in the 2 *Trpv1*^+^ populations, both of which contained peptidergic nociceptors (Figure 2, C and D). In the mouse, a third population, which did not express *Trpv1* but was positive for *Stx1a*, was observed (Figure 2D, bottommost row).

The murine transcriptomic associations suggested that staining for gene products from TRPV1^+^ neurons in human DRGs would yield a profile of neuronal perikarya and terminals consistent with nociceptors. Calcitonin gene-related peptide (CGRP), a well-known nociceptive neuropeptide (25, 26), served as a surrogate for TRPV1 itself, for which currently available antibodies did not yield effective labeling. In human DRGs, we observed numerous stained cells that varied in size and CGRP staining intensity (Figure 2E). Additionally, consistent with nociceptive afferent nerve terminations in superficial layers of the dorsal spinal cord, immunostaining showed that these layers were densely populated with CGRP-immunoreactive nerve endings (Figure 2F). The staining data in human DRGs, in combination with the 2 murine RNA-sequencing data sets (Figure 2, A–D) in which the nociceptive neuronal *Trpv1*^+^ populations were separated from other DRG cell populations, support the association of *Stx1a* with *Trpv1*^+^ nociceptive neurons in DRG. This has also been demonstrated in spatial transcriptomics from human DRGs, where expression of the genes encoding the CGRP precursors, *CALCA* and *CALCB*, overlaps that of *TRPV1* and tachykinin 1 (*TAC1*), which encodes the substance P precursor (27). This association was not seen with any of the other genes in the 7q11.23 WS locus, reinforcing *STX1A* as the candidate gene and the DRG as the site for the nociceptive deficiency seen in individuals with Dup7. Potential involvement of regions in the human CNS, and the exact colocalization of *TRPV1* and *STX1A* in human DRG neurons using multiplex fluorescence in situ hybridization, are explored in the next sections.

### Body-wide transcriptomics to consider spinal cord and brain as possible loci for STX1A-related analgesia.

**Figure 3:**
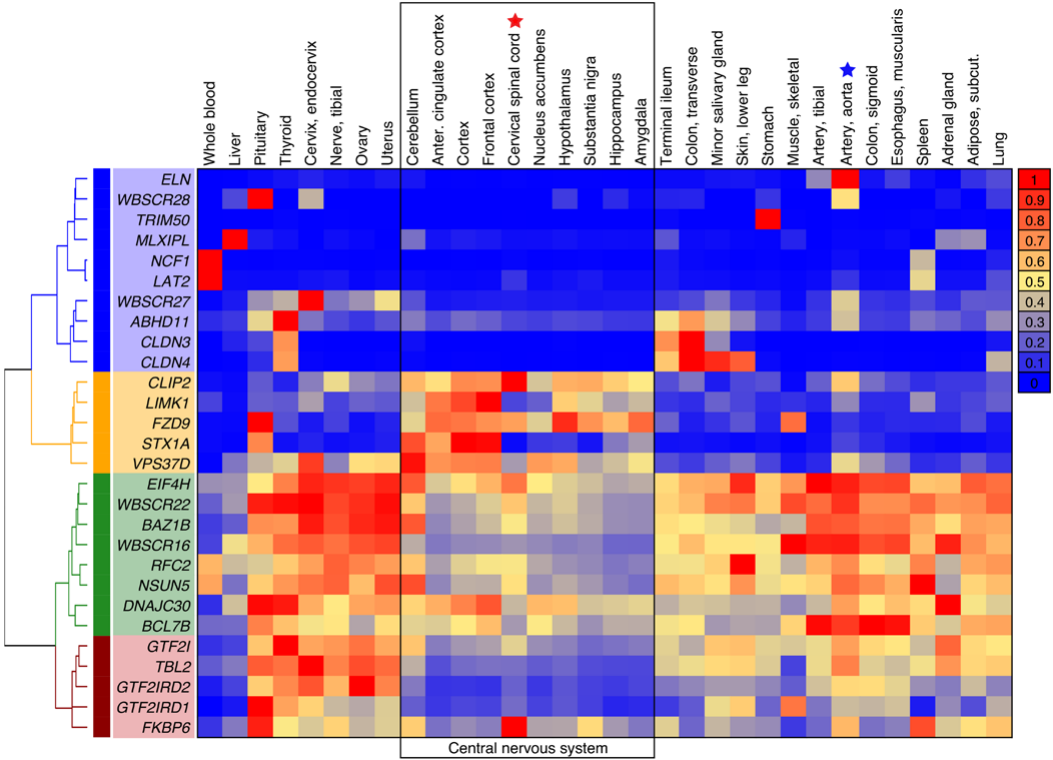
Expression of genes in the 7q11.23 locus in body organs and CNS. Transcript levels obtained from GTEx were analytically clustered for all the genes in the 7q11.23 WS/Dup7 locus as a heatmap according to relative expression. Enriched expression in multiple CNS regions is seen for CAP-Gly domain containing linker protein (*CLIP1*), Lim domain kinase 1 (*LIMK1*), frizzled class receptor 9 (*FZD9*), Syntaxin1A (*STX1A*), and vacuolar protein sorting 37 homolog D (*VPS37D*). Notably, in spinal cord (red star), *STX1A* was relatively not well expressed, though potential localization to a small subpopulation of spinal neurons cannot be ruled out. The aorta (blue star) strongly expresses elastin (*ELN*). Hierarchical clustering and heatmap visualizations were performed in R, clustered according to the ward.D2 method (70), and visualized with the heatmap.2 function.

If the spinal cord were involved in Dup7 pain insensitivity, then S*TX1A* or another gene in the 7q11.23 WS/Dup7 locus might be highly expressed in spinal neurons or in brain regions. Expression in CNS regions and body organs was determined using the GTEx transcriptomic resource (28) (Figure 3). Transcript levels were analytically clustered for all the genes in the WS/Dup7 locus as a heatmap according to relative expression in each of the CNS or body regions. Interestingly, *STX1A* expression was lower in spinal cord (red star in heatmap) than in 2 main forebrain regions, anterior cingulate and frontal cortex (Figure 3, bracketed section at top of the image). Four additional genes from the 7q11.23 WS/Dup7 locus showed strong expression in *CLIP2*, *LIMK1*, *FZD9*, and *VPS37D* whereas the others exhibited lower expression in CNS than in body regions or very high enrichment in specific tissues (e.g., *ELN* in aorta; Figure 3, row 1). These data draw focus to the DRG rather than the CNS as the site of STX1A action. The potential for higher CNS regions to contribute to a state of pain indifference is considered further in the Discussion.

### RNA sequencing of cell lines from participants with Dup7 or WS and typically developing individuals.

**Figure 4:**
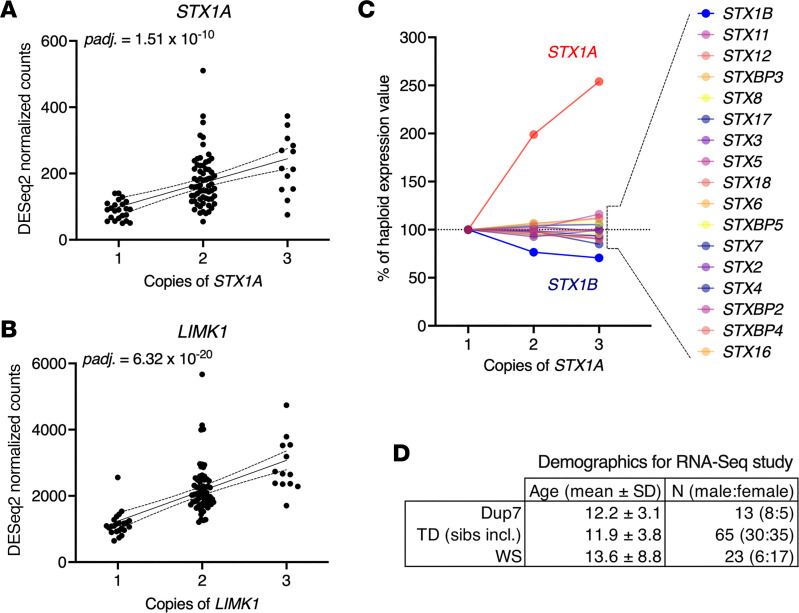
Gene dosage: RNA sequencing in human lymphoblastoid cell lines shows increasing *STX1A* transcript levels with increasing gene dosage. Data are derived from 13 Dup7, 65 typically developing, and 23 WS participants. (**A** and **B**) The *y* axes of **A** and **B** are in transcript counts for each gene from DESeq2 (adjusted *P* values, Benjamini-Hochberg). On the *x* axes, participants are grouped based on genomic copies. The significant linear relationship between copy number and transcript levels for *STX1A* and *LIMK1* is shown with 95% confidence limits for the linear regression (Prism 10, GraphPad). (**C**) Increasing transcript levels are specific to *STX1A*. The effect of gene dosage does not extend to any of the other syntaxin paralogs or syntaxin binding proteins as they are not in the CNV. The decrease in expression of *STX1B* was not statistically significant. (**D**) Demographics for lymphoblastoid cell line RNA sequencing.

We hypothesized that genes in the 7q11.23 CNV in individuals with Dup7 are overexpressed. To verify this, RNA was extracted from lymphocyte cell lines prepared from 23 children with WS, 40 typically developing children, and 13 children with Dup7 (Figure 4D). PolyA^+^ RNA libraries were sequenced on an Illumina NovaSeq 6000 to obtain a minimum of 49 million 150-base read pairs for each sample. A linear and highly significant (using DESeq2) gene dosage–dependent increase in the number of *STX1A* transcripts was observed across the 3 genotypes in nearly all the cell lines (Figure 4A). As a second example, we show cell line RNA-sequencing results for a more strongly expressed gene, *LIMK1*. In both cases transcript levels progressively increase with increasing gene dosage.

There are 21 other syntaxin paralogs or syntaxin binding proteins in the cell lines, and the comprehensiveness of RNA-sequencing allowed us to determine the specificity *STX1A* gene dosage compared withthese analogs. Only *STX1A* transcript levels were elevated. No effect of gene dosage was observed (*P* > 0.10) for any of the other 21 paralogs or binding proteins, none of which are located in the 7q11.23 WS/Dup7 locus (Figure 4, B and C). The specific and progressive increase in *STX1A* expression suggests that neurons from individuals with Dup7 have the genetic capacity to express more *STX1A* than do neurons from people with WS or typically developing controls.

### Overexpression of STX1A inhibits neuropeptide transmitter release from rat primary DRG neuronal cultures.

**Figure 5:**
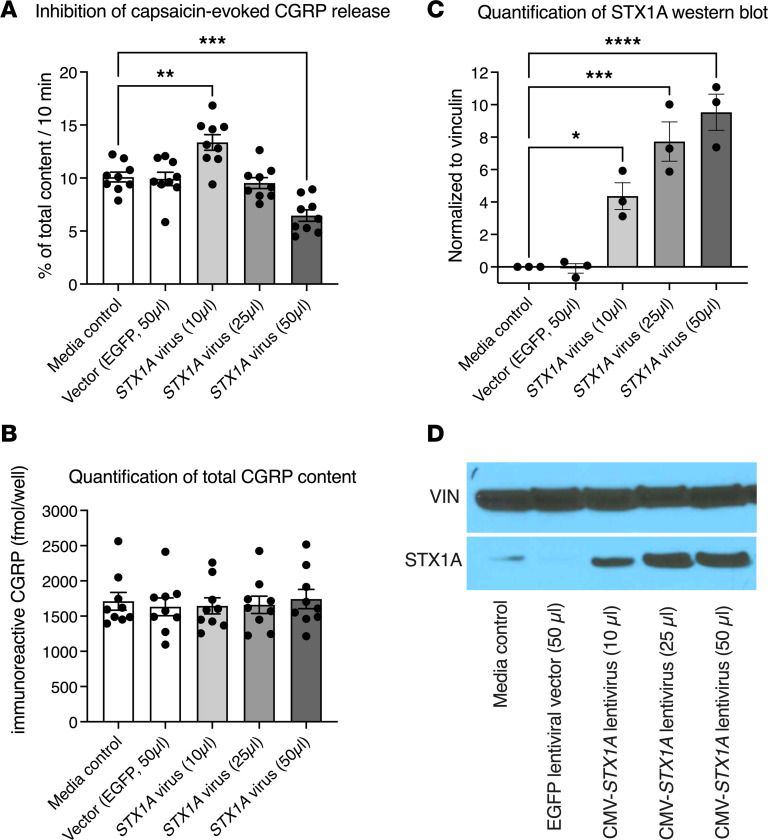
Overexpression of *STX1A* inhibits capsaicin-induced CGRP release from rat primary DRG neuronal culture. Capsaicin (30 nM) activates the TRPV1 ion channel, causing depolarization and calcium influx, which triggers neuropeptide release (CGRP) from the TRPV1-expressing neurons on the culture plate. The dose of capsaicin was chosen from a capsaicin dose and vector concentration pilot study (Supplemental Figure 1); 1 μL of virus preparation equals 1 × 10^5^ transducing units. Bars represent mean ± SEM and individual points are shown. (**A**) No difference is seen between control (culture medium, no vector) and vector only (expressing EGFP from the cytomegalovirus promoter). Ascending amounts of vector first produce an increase, and then a decrease, in capsaicin-evoked CGRP release (see also Supplemental Figure 1). (**B**) Addition of vector at any of the amounts used did not impair integrity of the primary cultured neurons. Loss of neurons from the plate would yield a decrease in total CGRP content. (**C**) Western blot analysis of STX1A content. Ascending amounts of vector produce significant, progressive increases in STX1A immunoreactive protein. (**D**) Photograph of the Western blot. Vinculin (VIN) was used to assess protein loading. Note that the basal amount of STX1A in the cultures is low. The increase from vector-generated STX1A may explain the initial increase in CGRP release with 10 μL vector. Release is then inhibited at 25 and 50 μL of vector. Statistics were performed by 1-way ANOVA followed by Dunnett’s post hoc test (GraphPad Prism 10) to test for differences in each group relative to the media control; *, **, ***, **** indicate significant differences from medium alone; *P < 0.05, **P < 0.01, ***P < 0.001, ****P < 0.0001; *n* = 9 primary cultures/condition.

Syntaxin1A is critical for fusion of large, dense-core synaptic vesicles with the plasma membrane and release of neurotransmitters and neuropeptides stored therein. As noted, TRPV1^+^ nociceptors in humans and rodents contain CGRP, which is stored in large, dense-core synaptic vesicles (29). To ascertain whether *STX1A* overexpression, as seen in the Dup7 cell lines, could functionally inhibit neuropeptide release, we caused primary cultures of DRG neurons to overexpress *STX1A* by transducing them with increasing concentrations of a lentivirus expressing *STX1A* (Figure 5 and Supplemental Figure 1; supplemental material available online with this article; https://doi.org/10.1172/jci.insight.176147DS1). A virus expressing EGFP served as the control. After 2 days of incubation, lentiviral vectors were removed and cells cultured for a further 5 days before conducting the release studies (see Supplemental Figure 1E). Release of CGRP from TRPV1^+^ neurons was triggered by addition of 30 nM (~the ED_50_) of capsaicin, a strong TRPV1 agonist (Supplemental Figure 1A). Released CGRP was measured in the incubation buffer with a specific and highly sensitive radioimmunoassay (30). *STX1A*-expressing lentivirus was evaluated over a 50-fold range of viral transducing units. We observed a concentration-dependent but biphasic effect: low doses of *STX1A* expressing viral vector caused an enhancement of capsaicin-evoked Trpv1-mediated CGRP release whereas higher levels of vector inhibited CGRP release (Figure 5A and Supplemental Figure 1A). This is consistent with Western blot analysis of STX1A protein content in the cultures. Low initial *STX1A* levels increased progressively as the amount of vector increased (e.g., Figure 5, C and D, and Supplemental Figure 1, C and D). Thus, the primary cultures appeared to make a sufficient, but apparently suboptimal, quantity of syntaxin1A, such that release was enhanced by supplementing the *STX1A* levels at low concentrations of lentiviral expression vector and then inhibited as overexpression occluded the vesicle fusion machinery. Taken together, the biphasic effect is consistent with the hypothesis that a broad range of syntaxin1A can support vesicle fusion and peptide release (e.g., in WS and diploid individuals) but that exceeding this range is inhibitory to the release process (e.g., as in individuals with Dup7).

It is important to note that the decreased release seen with higher amounts of viral vector was not due to viral toxicity toward neurons in the culture since the basal content of CGRP in the cultures was not altered by increasing viral concentrations (Figure 5B). If excessive viral vector was killing DRG neurons, the basal level of CGRP would have decreased due to loss of cells from the culture plate. This did not occur (Figure 5C), indicating preservation of the integrity of the primary cultures.

Table 2 shows that a parallel can be drawn between the culture experiments and gene dosage and pain sensitivity status in humans: 1 or 2 copies of the *STX1A* gene in individuals with WS (+/–) or their siblings (+/+), respectively, supports adequate nociceptive functions, whereas 3 copies are inhibitory. Although the effects of a full *STX1A* knockout in humans are currently unknown, it likely would involve other severe phenotypic presentations (31). However, extrapolating to include the *Stx1a*-knockout mouse, which exhibits an increased nociceptive phenotype (32), then nociceptive responsiveness ranges from enhanced sensitivity with 0 copies (mouse), to normal with 1 or 2 copies, and inhibited with 3 copies (Table 2). Taken together, the release and expression data support an *STX1A* overexpression mechanism for nociceptive dysfunction in individuals with Dup7.

### STX1A is coexpressed with TRPV1 in human DRG neurons.

**Figure 6:**
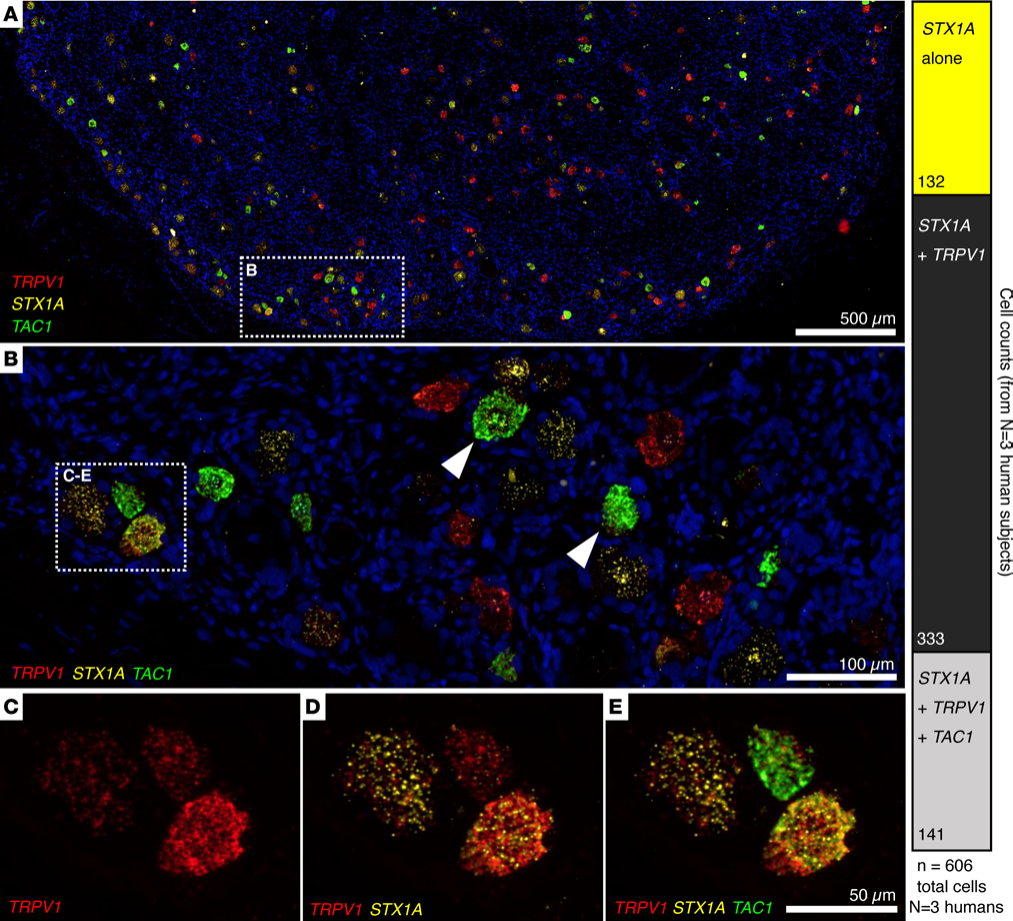
Human DRG multiplex in situ hybridization for nociceptive markers and *STX1A*. (**A**–**E**) Human DRG sections analyzed using 3-plex RNAScope in situ hybridization with probes for *TRPV1*, *STX1A*, and *TAC1* (preprotachykinin), which codes for the precursor for substance P, a neuropeptide that colocalizes with TRPV1 in DRG neurons. Hybridization was conducted with formalin-fixed, paraffin-embedded 6 μm sections of L4 human DRGs (*n* = 3 ganglion sections from *n* = 3 different individuals). In situ signals show a high degree of colocalization between *STX1A* and *TRPV1* (**B** and **E**). Additionally, all *TAC1*^+^ cells were found to be co-positive with *STX1A* and *TRPV1*. The bright green hybridization signal is for the *TAC1* transcript (arrowheads in **B** point to 2 large *TAC1*^+^ neurons) such that the bright *TAC1* signal often obscures the other labels, as can be appreciated in the colocalization overlays in **C**–**E**. The bar graph quantitates the cellular coexpression counts. Most neurons coexpress *STX1A* and *TRPV1*, although approximately 20% of *STX1A* signal can be found in *TRPV1*-negative neurons. The combinatorial colocalization matrix is analyzed further in Figure 7.

While the above studies demonstrated transcriptomic and functional associations between *STX1A*^+^ and *TRPV1*^+^ nociceptive DRG neurons, they presuppose *STX1A* is coexpressed with *TRPV1*. We explicitly tested coexpression in human DRG neurons using multiplex fluorescent in situ hybridization. Data were derived from 6 individual human lumbar DRGs (3 for the triplex stain and 3 for the quadruplex stain). Formalin-fixed, paraffin-embedded DRG sections were simultaneously hybridized with RNAScope probes for *TRPV1*, *STX1A*, and *TAC1* in 1 set of studies. The *TAC1* gene encodes the substance P precursor, a neuropeptide highly expressed in nociceptive DRG neurons along with *TRPV1* and the genes coding for CGRP (25, 26). Human DRGs contain many *TRPV1*-expressing neurons, seen as red cells in the panoramic view of DRG in panel A of Figure 6. We counted *TRPV1* hybridization signals in 463 neurons in 3 sections from 3 individuals. Among these neurons, *STX1A* was expressed in *all* the *TRPV1*-expressing neurons though to a variable extent (see also Supplemental Figure 2). The *TAC1* transcript was expressed in approximately 50% of the *TRPV1*^+^*STX1A*^+^ neurons. Additionally, approximately 20% of *STX1A*-expressing neurons were in a distinct population that did not contain either *TRPV1* or *TAC1* transcripts (Figure 6, bar on right). The latter are possibly equivalent to the nonpeptidergic population delineated in mouse DRGs (Figure 2D). Importantly, the multiplex in situ hybridization showed a complete overlap in *STX1A* expression with *TRPV1*, implicating *STX1A* in all nociceptive sensations mediated by *TRPV1* neurons, such as heat and inflammation.

**Figure 7:**
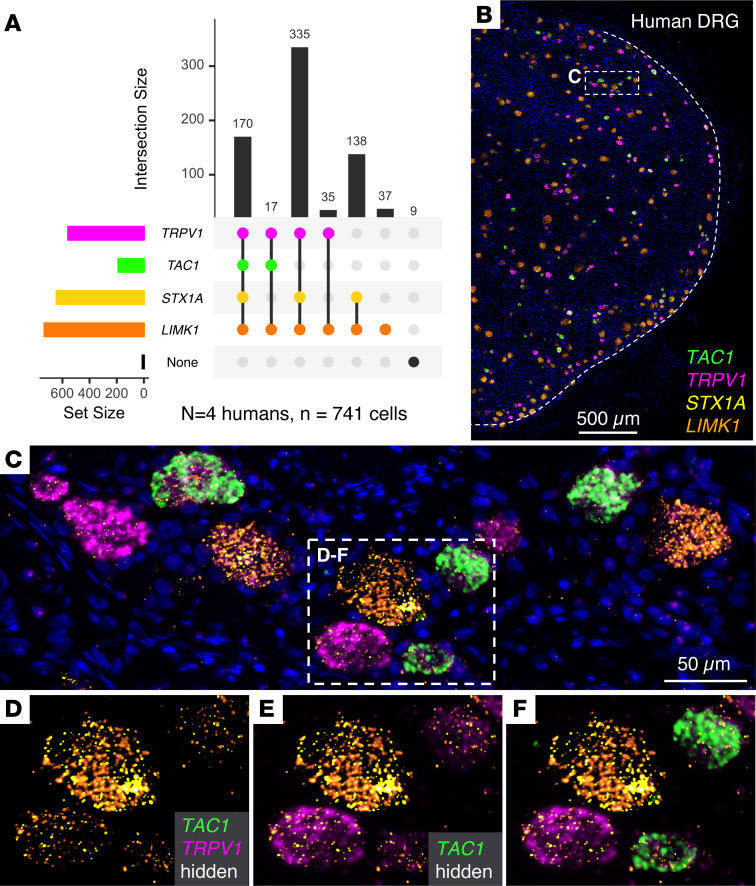
Analysis of *TRPV1, STX1A, LIMK1,* and *TAC1* expression in human DRG using multiplex fluorescence in situ hybridization. (**A**) Quantitative cell count plot showing combinatorial coexpression patterns for the 4 transcripts. The majority of *TRPV1*^+^ neurons (90%) coexpress *STX1A*. A subpopulation of *STX1A*^+^*LIMK1*^+^ expressing neurons (18% of total neurons) is also present. Only 6% of *TRPV1*^+^ neurons express *LIMK1* without *STX1A*. These data are consistent with the counts in Figure 6. Numbers of labeled cells were obtained by counting *n* = 3 ganglion sections from *n* = 3 different individuals. (**B**) Panoramic photomicrograph of a portion of the L4 ganglion simultaneously hybridized with a 4-plex probe set consisting of *TRPV1*, *STX1A*, *LIMK1*, and *TAC1* shows intermingling of the different neurons in the ganglion. (**C**) Higher magnification of region designated in **B** showing a range of expression for each gene in the different neurons. *TRPV1* and *STX1A* are expressed to varying degrees in all the neurons in this field, but their very strong signals tend to bleed and coalesce. (**D**–**F**) Successive visualizations of the 4-plex in situ. (**D**) *LIMK1* and *STX1A* coexpression in 4 neighboring neurons, each expressing a range of transcript for the 2 genes. (**E**) Overlay of the magenta signal for *TRPV1* shows it is expressed in all 4 neurons but not abundantly in the large diameter neuron in the upper center left. (**F**) Overlay of the fourth *TAC1* signal shows abundant hybridization in the 2 neurons on the right side.

One additional gene in the 7q11.23 locus, *LIMK1*, was examined in knockout mice, and it was observed that gene knockdown or deletion partially inhibited hyperalgesic responses (33, 34). To examine the relationship of *STX1A* to *LIMK1* as well as *TRPV1* expression, a second set of hybridization experiments were conducted using a “4-plex” gene combination of *LIMK1*, *TRPV1*, *STX1A*, and *TAC1* (Figure 7). In L4 ganglia, 714 neurons, obtained from 3 human organ donors (different from those in the above 3-plex study), were observed to have coexpression of the 4 transcripts. The majority of *TRPV1*^+^ neurons (90%) coexpressed *STX1A*, which is consistent with the counts presented in Figure 6. We also observed that coexpression of *STX1A* and *LIMK1* was a common feature of *TRPV1*^+^ neurons, with 90% of *TRPV1*^+^ neurons containing all 3 genes (Figure 7A). A panoramic image of a portion of the L4 ganglion shows the intermingling of the different neurons in the ganglion (Figure 7B). In an enlarged inset, the expression level of all 4 genes is seen to be variable (Figure 7, C–F). Analysis of the fluorescent signal intensity in the context of colocalization of *STX1A*, *LIMK1*, and *TRPV1* in human DRGs showed that high-*TRPV1*-expressing neurons colocalized with all levels of *STX1A* expression. This observation is notable because it duplicates the observation in Figure 6 that nociceptive responses in neurons with all levels of *TRPV1* expression can be modulated by *STX1A* overexpression. In contrast, neurons with high *LIMK1* expression were a distinct population from those exhibiting high *TRPV1* expression (Supplemental Figure 2), making it difficult for *LIMK1* overexpression to impact nociceptive functions of high *TRPV1*-expressing neurons. Since *TRPV1*^+^ neurons transduce a broad spectrum of nociceptive insults (22, 35), the broad spectrum of pain deficiencies seen in individuals with Dup7 could be mediated by *STX1A* overexpression in this neuronal population.

## Discussion

**Figure 8:**
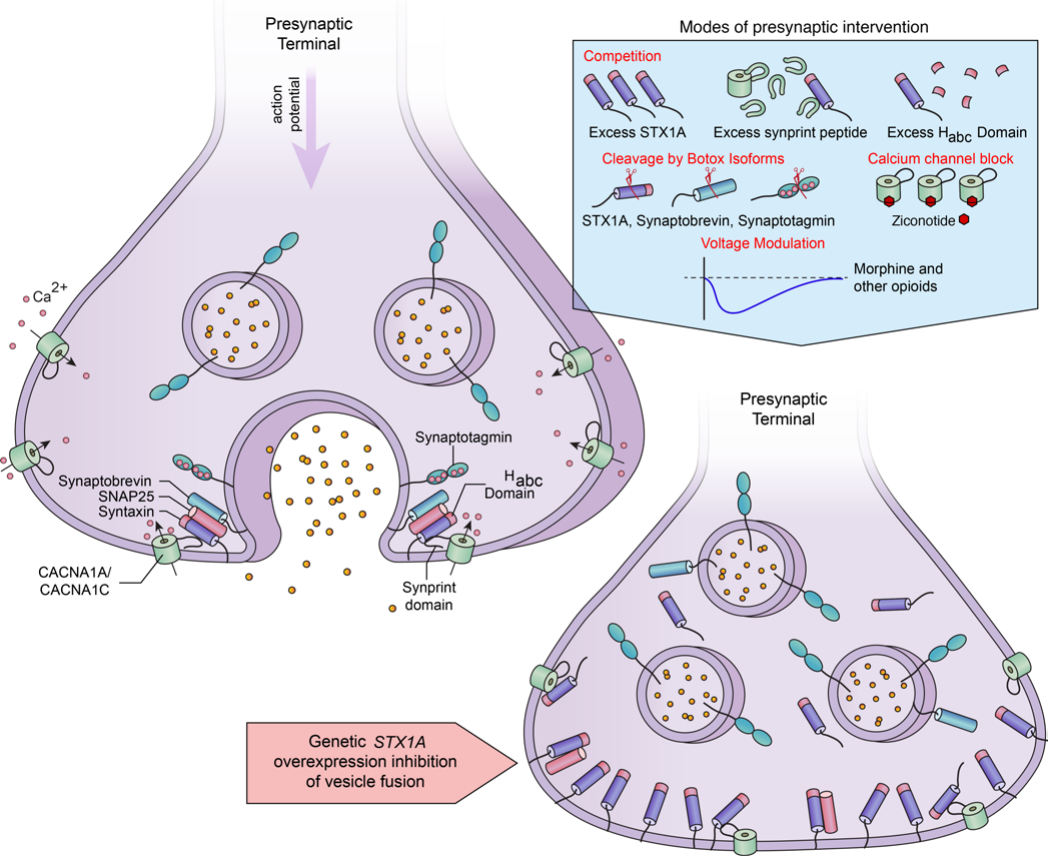
Summary and translational mechanisms for nociceptive presynaptic inhibition. Upper left: a simplified schematic of vesicle fusion showing SNARE complex and the tethered calcium channel. Depolarization of the terminal causes an influx of calcium through voltage-gated calcium channels and initiates vesicle fusion. Lower right: overexpression of *STX1A* interferes with the fusion machinery through multiple interactions, which occlude fusion and interrupt neuropeptide release from primary afferents. The resulting phenotype is profound analgesia. The blue panel depicts multiple mechanisms for inhibition of presynaptic vesicle fusion ranging from competition, to disruption of STX1A-calcium channel tethering (52, 76), to enzymatic cleavage with botulinum toxins (59). Three interventions, botulinum toxin, morphine, and the calcium channel blocker ziconotide (77, 78), are currently used therapeutic agents. Intrathecal administration of morphine or ziconotide produces potent and effective analgesia.

Here we describe a highly effective inhibition of nociceptive events occurring in many parts of the body in individuals with Dup7. While a high pain tolerance was previously observed in some individuals with Dup7 (15), it was not thought to be central to the syndrome, and there was no clear mechanism to account for their altered pain sensitivity. We identify *STX1A* as the candidate gene in the 7q11.23 WS/Dup7 locus, and the DRG as the likely nervous system site mediating the nociceptive insensitivity. In Dup7-derived cell lines, overexpression was verified by RNA sequencing, and a gene dosage effect was evident when haploid individuals with WS and diploid individuals were included. Virally mediated overexpression of *STX1A* in vitro inhibited stimulus-evoked neuropeptide release from nociceptive DRG neurons, implicating the synaptic vesicle fusion process. Coexpression of *STX1A* and *TRPV1* was demonstrated in human DRG neurons, providing evidence nociceptive signals can be modulated by *STX1A* overexpression. Together these data indicate that the pain insensitivity is due to *STX1A* overexpression, causing a dominant-negative SNAREopathy. Beyond this, the mechanism points to the primary afferent SNARE complex as a node of vulnerability for nociceptive processes that can be exploited for development of analgesic agents (Figure 8).

We attribute the broad impairment of nociceptive function in people with 7q11.23 duplication syndrome to overexpression of *STX1A* in the TRPV1^+^ subpopulation of peripheral nociceptive sensory neurons. We focus on this population because the TRPV1^+^ neurons are critical mediators of multiple nociceptive modalities, and inhibition of this population can account for the multiple types of pain insensitivity presented by the individuals with Dup7. TRPV1^+^ DRG neurons are found in every sensory ganglion from trigeminal to sacral ganglia. Furthermore, animal and human studies showed that TRPV1^+^ neurons generate nociceptive signals from many tissue damage conditions, including noxious heat, cancer, osteoarthritis, surgical incision, inflammation, and other stimuli. Conversely, *blocking* TRPV1^+^ neuronal activity using the highly potent TRPV1 agonist resiniferatoxin produces a wide range of analgesic actions that parallel the range of insensitivity seen in individuals with Dup7 (1, 20, 22, 36, 37). Thus, the TRPV1^+^ neurons fulfill the operative mechanisms of wide distribution in the body, responsiveness to multiple types of nociceptive stimuli as seen in people with Dup7, and analgesia upon effective inhibition. The fact that the *TRPV1* and *STX1A* transcripts are coexpressed in the same DRG neurons further supports the hypothesis that *STX1A* overexpression in the TRPV1^+^ population of DRG neurons is the basis of the strong pain insensitivity seen in individuals with Dup7.

### Increasing STX1A expression with increasing 7q11.23 CNV dosage.

A fundamental assumption is that duplication leads to overexpression of *STX1A*. Indeed, we detected progressive levels of expression by RNA sequencing of cell lines generated from individuals with +/–, +/+, and +/+/+ genotypes. In +/+/+ individuals, the elevation in *STX1A* transcript occurred without affecting expression of other syntaxin analogs not contained within the duplication/deletion boundaries (Figure 4C), a result that serves as an important control, indicating that *STX1A* expression is specifically modulated among syntaxin gene family members. Some of the family members have a strong sequence homology at the amino acid level (e.g., STX1B) and could, if expressed in the same neuron, be capable of compensating for each other to some extent. However, this consideration is more applicable to a *loss*-of-function mutation, where a similar family member could substitute for a gene loss, rather than the overexpression situation seen with 7q11.23 WS locus duplication, since additional syntaxins would only add to the occlusion of fusion already present.

### STX1A overexpression attenuates neuropeptide release from nociceptive neurons.

We observed that, in primary cultures, overexpression of *STX1A* was sufficient to attenuate peptide release induced by the TRPV1 agonist capsaicin (Figure 5). Capsaicin produces an influx of calcium (38) that normally triggers neuropeptide release from synaptic vesicles, but this release was impaired by *STX1A* overexpression. Conceptually, it is possible that overexpression could lead to either inhibition or enhancement of release depending on the basal level of syntaxin expression. Our in vitro lentiviral vector *STX1A* expression experiments did display an initial increase in capsaicin-evoked CGRP release from TRPV1^+^ neurons (Figure 5 and Supplemental Figure 1). However, higher levels of STX1A protein inhibited capsaicin-induced release, consistent with overexpression as the mechanism for impairing presynaptic vesicle fusion and analgesia (Figure 5). The overexpression of *STX1A*, coupled with functional impairment of evoked release, is consistent with a “dominant-negative synaptopathy” or more precisely a SNAREopathy (39–41), discussed further below (Figure 8). The proposed mechanism is molecularly distinct from that of the several loss-of-function gene mutations causing pain insensitivity, which range from pain channelopathies to other mutations that compromise DRG function or integrity (6, 7, 42, 43).

### Mechanisms for inhibition of vesicle fusion by STX1A overexpression.

The presynaptic vesicle fusion apparatus is a massive macromolecular nanodomain involving proteins integral to the vesicle membrane, to the presynaptic ending, and to the synaptic cytosol (18). STX1A protein is present in large, dense-core vesicles in nerve endings of peptidergic neurons and peptide hormone-secreting cells (44, 45). The function of STX1A in vesicle fusion is well known and understood from multiple studies of vesicle docking in adrenal chromaffin or pancreatic β cells (46). STX1A contains both a SNARE domain and an H_abc_ domain. The latter interacts with syntaxin binding protein 1 (STXBP1, MUNC18-1) to hinder SNARE complex formation, vesicle docking, and release (47, 48). Overexpression of *Stx1A* inhibits dense-core vesicle release of insulin by pancreatic β cells (49, 50), and overexpression of just the region containing the Stx1A H_abc_ domain competitively interferes with clustering of the vesicular release machinery at the plasma membrane and successful docking of synaptic vesicles (51). Experiments in mice overexpressing *Stx1A* by 30% in pancreatic β cells, and in β cell lines in culture, demonstrated significant reduction in insulin secreted by these cells (50, 51). These studies demonstrate that excess STX1A protein or fragments thereof interfere with vesicle fusion.

Vesicle binding is triggered by presynaptic calcium influx, and the actions of STX1A are amplified, tethering voltage-gated Ca^2+^ channels in the complex via the Ca^2+^ channel’s synaptic protein interaction (synprint) domain (52–54). Ostensibly, the macromolecular tethering optimizes spatial coupling between channel-mediated presynaptic calcium influx, a requisite trigger for vesicle exocytosis, and docking of the vesicle itself, which can occur within 200 μs of calcium influx (55). Also, repolarization can be facilitated by binding to and positioning of K^+^ channels (56). Thus, there is a temporal advantage to preassembly of components needed for the multiple steps in vesicle docking. Each of these steps may be vulnerable to inhibition by an excess of STX1A protein (Figure 8).

### Direct mutation of STX1A and human brain regions.

Recently, mutations in vesicle fusion proteins have been designated as SNAREopathies (39), referring to the SNARE domain common to several vesicle fusion proteins, including STX1A. SNAREopathies are a distinct subset of synaptopathies. Heterozygous loss-of-function or missense mutations of genes encoding proteins in this synaptopathy subgroup (e.g., *STXBP1* and *STX1A*) can cause diverse neurological and developmental disorders (39). Mutations in genes mediating the synaptic docking process, including *STX1A*, are rare. Individuals harboring mutations in *STX1A* have not been carefully studied for alterations in pain sensitivity, though it is interesting to note that different phenotypes occur if the mutations involve interaction with STXBP1, which normally inhibits STX1A-SNARE interactions, or the domain of STX1A that interacts directly with the other 2 members of SNARE complex (31). This raises the possibility that the inhibitory effects of overexpression of STX1A protein may vary based on genetic variation in other interacting SNARE proteins.

### Stx1a and Limk1 in mouse models.

Overexpression of *STX1A* in individuals with Dup7 is related to inhibition of nociception, which contrasts with pain hypersensitivity from *Stx1a*-knockout mice (Table 2). In the basal state, these mice do not exhibit altered synaptic activity in dorsal spinal cord neurons. However, after peripheral nerve injury, spinal neurons exhibit increased amplitude of evoked EPSC and increased behavioral mechanical allodynia (32), suggesting a role for Stx1a in nociceptor plasticity and hyperalgesia in conditions of neuropathic pain. Thus, with the caveat that the knockout is in the mouse, 0 copies appear to enhance pain mechanisms; in people 1 to 2 copies support normal pain processing, and 3 copies cause inhibition (Table 2). This pattern implies a delicate balance of proteins involved in presynaptic vesicle fusion.

### Limitations.

One limitation relates to the potential role of other genes within the CNV in modulation of pain sensitivity. The only encoded protein within the CNV aside from STX1A that has previously been investigated in pain is LIMK1 (33, 34). The *LIMK1* gene is well expressed in both pain-sensing and non-pain-sensing DRG neurons but is not enriched in the mouse *Trpv1* linage neurons, as is seen with *Stx1a* (Figure 2). This expression profile suggests a general kinase function for *LIMK1* in DRG neurons rather than a specific association with nociceptive processes. However, in the mouse, LIM kinase activity promotes the development of nerve injury and inflammatory hyperalgesic responses (33, 34). Because of its potential role in *hyper*algesia, it is unlikely that overexpression of *Limk1* would yield analgesia; additional murine studies may provide further understanding of potential interactions. Another potential limitation that is being evaluated is the stability of the phenotype with age. Additionally, other sensory domains can be investigated. For example, we did not receive reports on cold pain tolerance. Finally, the neurons that express *STX1A* in cortical areas need to be mapped. *STX1A* is expressed in several brain areas, and this anatomical information would help assess its function in these areas.

### Multiple potential routes to analgesia.

The insensitivity to diverse nociceptive stimuli seen in individuals with Dup7 provides genetic validation for what we believe are potentially novel routes to analgesia based on STX1A that may be actionable in several ways (Figure 8, upper right panel). First, the fusion release process from *TRPV1*-expressing DRG neurons appears particularly susceptible to modulation by multiple vesicle-directed treatments. Indeed, several known, proposed, or potential analgesic manipulations, including morphine, calcium channel blockers (ziconotide), and experimental peptides, and other molecules (57), converge on the process of presynaptic primary afferent vesicle fusion (Figure 8). In vitro experiments with several peptidomimetics show that providing excess H_abc_ domain of STX1A or the calcium channel “synprint” sequence can inhibit vesicle fusion (51, 52, 54, 58). Such a protein-protein interaction mechanism could be adapted to yield analgesia (58). A well-known enzymatic approach is cleavage of SNARE complex proteins by serotypes of the light chain of botulinum toxin. Hydrolysis of the SNARE proteins causes long-duration presynaptic inhibition of neurotransmission (59). Ligand-targeted botulinum light chain can inhibit experimental nociceptive stimuli via agonist-mediated uptake into spinal cord second-order neurons (60). Inhibition of CGRP release by in vitro lentiviral overexpression of *STX1A* (Figure 5) suggests the possibility of an in vivo gene therapy approach to analgesia (61–65). Last, it is notable that intrathecal or epidural administration of morphine, other opioids, and the calcium channel blocker ziconotide can inhibit presynaptic release of transmitter from terminals of primary afferents in the dorsal spinal cord, which is an optimal site for producing analgesia (58) and is the same site at which an overabundance of STX1A is hypothesized to act. While opioids remain the most effective analgesics in the pharmacopeia, opioid analgesia is accompanied by many deleterious side effects (e.g., addiction, CNS and respiratory depression), and finding effective alternatives is a priority objective. The several mechanisms outlined support the idea that manipulation of STX1A or associated processes in DRG neurons (66) has the potential to yield new types of analgesic agents.

## Methods

### Human participants.

Individuals with WS or Dup7; their unaffected, typically developing siblings; and unrelated, typically developing individuals participated in our longitudinal WS and Dup7 neurogenetics research program (protocol 10M0112/NCT01132885) (67, 68).

Demographic characteristics are reported in Table 1. The mean ± SD age of the 38 child participants (15 female, 23 male, Table 1) was 8.45 ± 3.97 years. Two adults with Dup7 also participated. The behavioral pain sensitivity data are derived from these 40 individuals, of whom 11 had the Dup7 +/+/+ genotype, 13 had the WS +/– genotype, and 16 were typically developing (+/+). The sex distribution was approximately equal for all groups except the WS +/– cohort (Table 1). The lymphoblastoid cell line RNA-sequencing results were derived from a larger cohort of 101 participants composed of 13 individuals with Dup7, 65 typically developing individuals, and 23 individuals with WS (Figure 4D).

### Pain sensitivity assessments.

A 7-point numeric rating scale was provided to the parents and the 2 adults. The scale showed all 7 numbers, but only 3 (numbers 1, 4, 7) had anchor points with the descriptions provided below (except that rather than “the participant’s” it said “the child’s” or “your” age, depending on whether the conversation was with the parent or the adult participant). Parents/adult participants and partners were shown the scale on an 8½″ × 11″ piece of paper (landscape orientation), with the numbers from 1 to 7 equally spaced across the page and the text provided below the anchor points (numbers 1, 4, 7). The rating scale was as follows: 1: Seems extremely insensitive to pain; 2; 3; 4: Seems to feel pain at the level expected for the situation and the participant’s age; 5; 6; 7: Seems extremely sensitive to pain.

Parents also provided narrative accounts of illnesses or events that they or treating physicians judged should have elicited a painful response but to which the child displayed no negative reaction. The total number of insensitivity events was tabulated for the participants with Dup7 and their sibs, as well as participants with WS and their sibs (Table 1).

### Human DRG recovery and processing.

Lumbar DRGs were obtained from organ donors (*n* = 3) within 2 hours of aortic cross clamp (AnaBios Corp) or during stat autopsy (*n* = 3) at the NIH National Cancer Institute’s Laboratory of Pathology. See Supplemental Methods.

### Immunohistochemical staining for CGRP in human DRG and spinal cord.

For immunohistochemistry, 6 μm sections were deparaffinized and hydrated through graded alcohols to distilled water, followed by antigen retrieval using pH 6 citrate buffer at 70°C for 40 minutes. Primary rabbit polyclonal anti-CGRP antibody for immunohistochemistry was from Peninsula Laboratories (T-4239). See Supplemental Methods.

### In situ hybridization.

The RNAScope method (Advanced Cell Diagnostics) was used for in situ hybridization analysis of *STX1A*, *TRPV1*, *TAC1* (substance P precursor), and *LIMK1*. RNAScope Multiplex Fluorescent assays v2 (Advanced Cell Diagnostics) with Tyramide Signal Amplification (Opal Reagent Systems; PerkinElmer) were used for multiplex in situ hybridization as described previously (69). See Supplemental Methods.

### Visualization of RNA-sequencing data using heatmaps and scatterplots.

Hierarchical clustering and heatmap visualizations were performed on expression values (median RPKM, GTEx Analysis v6) (28) for all 7q11.23 WS locus genes in R. Data were scaled in R, clustered according to the ward.D2 method (70), and visualized with the heatmap.2 function.

### Transcriptomic analysis of DRG neuronal subtypes expressing Stx1A.

Transcriptomic analyses in Figure 2, A and B, were performed using the data from Goswami et al. 2014 (23) and Zheng et al. 2019 (24). The physiologically characterized categories of DRG neurons were described (71, 72). See Supplemental Methods.

### Lymphoblastoid cell lines and RNA sequencing.

RNA was extracted from lymphocyte cell lines from 23 children with WS, 40 typically developing children, and 13 children with Dup7. RNA sequencing was performed at the NIH Intramural Sequencing Center. See Supplemental Methods.

### Cell culture.

DRGs were dissected from all spinal levels of adult male (150–175 g) Sprague-Dawley rats (Envigo), and the cells were dissociated as previously described (73, 74). See Supplemental Methods.

### Overexpression of STX1A.

Lentiviral constructs containing a) the CMV promoter, human *STX1A* (National Center for Biotechnology Information [NCBI] accession BC064644), internal ribosome entry site (IRES), and EGFP or b) CMV, IRES, and EGFP were used to enhance STX1A protein expression in neuronal cell cultures, as previously described (74). See Supplemental Methods.

### Immunoblotting.

Tissues or cells were harvested, lysed in RIPA buffer (Santa Cruz Biotechnology), sonicated, and cleared of cellular debris by centrifuging at 4,000 rpm for 2 minutes. The protein concentration in lysates was quantified using Lowry assay. Protein aliquots were electrophoresed in a 12% SDS-PAGE and transferred to a PVDF membrane. See Supplemental Methods.

### Measurement of CGRP.

CGRP content was measured in both the culture media and the cells on the culture plate to assess, respectively, peptide release and integrity of the culture after stimulation. See Supplemental Methods.

### Statistics.

Age data are mean ± SD. Difference in pain sensitivity ratings was determined using Kruskal-Wallis testing in Prism 10 (Figure 1, GraphPad). Statistics for the human cell line gene dosage study (Figure 4) were calculated using DESeq2 in R. For DESeq2 analyses, adjusted *P* values are reported using the Benjamini-Hochberg method (default). For release studies (Figure 5 and Supplemental Figure 1), data are expressed as the mean ± SEM from at least 3 replicates of each experiment. Differences in *STX1A* expression and CGRP release in DRG cultures were determined using 1-way ANOVA and Dunnett’s post hoc test to test for differences versus a media control. Significant differences (*P* < 0.05, brackets) are shown for each experiment. In all cases, significance was established as *P* < 0.05, comparing vector-treated controls with *STX1A* construct–treated experimental groups.

### Study approval.

Study procedures for human participants were approved by the NIH IRB. Adults and parents of minor participants provided written informed consent and children provided assent. For stat autopsies, the standard autopsy consent includes language permitting scientific use of tissues removed.

For photographs used, separate informed consent was obtained for use of these images and has been retained by study investigators. For primary cell lines, the study was approved by the animal care and use committee of the Indiana University School of Medicine.

### Data availability.

RNA-sequencing data from human DRGs, mouse DRGs, and GTEx are publicly available from the NCBI Sequence Read Archive (BioProject PRJNA308243) and previous publications (22–24, 28, 75). NIMH data on rare 7q11.23 participants are not publicly available due to institutional (IRB) restrictions. All other data supporting the findings are available within the article and supplement. Individual values from display items are found in the Supporting Data Values file.

## Author contributions

MJI, CBM, MDG, and KFB conceived the study; MDG, DPE, TAN, MHG, JSK, CBM, and KFB were responsible for clinical elements; AJL, MRS, and DM were responsible for fluorescence imaging and analysis; JCF and MRV were responsible for primary cultures, lentivirus, and peptide assays; MJI, MRS, MHG, and MDG were responsible for transcriptomic and other data analyses; AJM, MJI, and KFB supervised; MJI was responsible for writing the original draft; MJI, MRS, CBM, AJM, JSK, MDG, and KFB were responsible for revising the manuscript.

## Supplementary Material

Supplemental data

Unedited blot and gel images

Supporting data values

## Figures and Tables

**Table 1:**
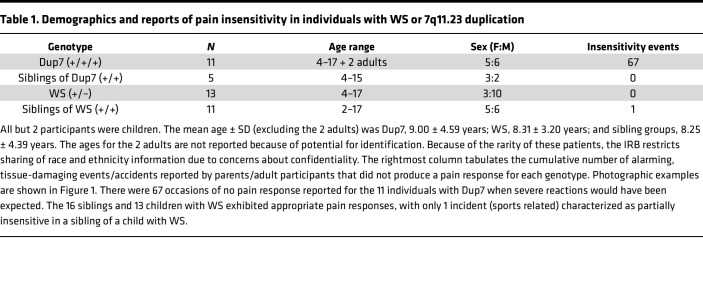
Demographics and reports of pain insensitivity in individuals with WS or 7q11.23 duplication

**Table 2:**
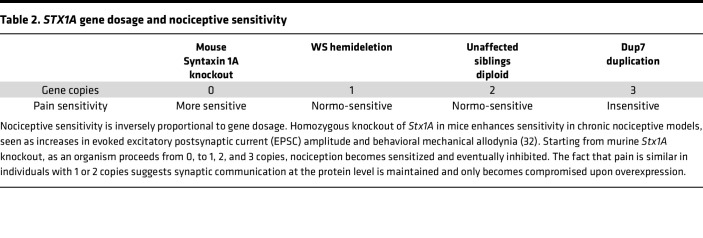
*STX1A* gene dosage and nociceptive sensitivity

## Version 1 01/23/2024

In-Press Preview

## Version 2 02/22/2024

Electronic publication
