# Supplementary material for: Syntaxin1A overexpression and pain insensitivity in individuals with 7q11.23 duplication syndrome
Source: JCI Insight. 2024 Feb 22;9(4):e176147. doi: 10.1172/jci.insight.176147 (PMC10967379; doi:10.1172/jci.insight.176147)
Supplement: Supplemental data [file jciinsight-9-176147-s216.pdf]

Supplementary Materials: Syntaxin1A overexpression and pain insensitivity in individuals with 7q11.23 duplication syndrome

Michael J. Iadarola[1*], Matthew R. Sapio[1], Amelia J. Loydpierson[1], Carolyn B. Mervis[2], Jill C. Fehrenbacher[3], Michael R. Vasko[3], Dragan Maric[4], Daniel P. Eisenberg[5], Tiffany A. Nash[5], J. Shane Kippenhan[5], Madeline H. Garvey [5], Andrew J. Mannes[1], Michael D. Gregory[5†], Karen F. Berman[5†]

Author Affiliations

1. Department of Perioperative Medicine, Clinical Center, National Institutes of Health, Bethesda, MD 20892
2. Neurodevelopmental Sciences Laboratory, Department of Psychological and Brain Sciences, University of Louisville, Louisville, KY, 40292
3. Department of Pharmacology & Toxicology, Indiana University School of Medicine, Indianapolis, IN 46202
4. Flow and Imaging Cytometry Core Facility, National Institute of Neurological Disorders and Stroke, National Institutes of Health, Bethesda, MD 20892
5. Clinical and Translational Neuroscience Branch, National Institute of Mental Health, National Institutes of Health, Bethesda, MD 20892

[†]These authors contributed equally

*To whom correspondence should be addressed:
Department of Perioperative Medicine,
Building 10 Room 3D56, Clinical Center
National Institutes of Health,
10 Center Drive
Bethesda, MD 20892
e-mail Michael.iadarola@nih.gov

Supplementary Materials and Methods.

Human DRG Recovery and Processing

Lumbar DRGs were obtained from organ donors (N=3) within 2 hours of aortic cross clamp (AnaBios Corp, San Diego, CA) or at “stat” autopsy at the Laboratory of Pathology, NCI, NIH. The L4 or L5 ganglia, bilaterally, and lumbar spinal cord were recovered from these donors after opening the vertebral canal. Tissue was frozen on dry ice and stored frozen at -80˚C until used for immunocytochemistry or *in situ* hybridization. One ganglion from the respective vertebral segment was immersion fixed in 4% neutral buffered formalin for 18 to 24 hours and embedded in a paraffin block. Six μm sections were cut and mounted by Histoserv Inc (Germantown, MD) using RNase free procedures. Slide mounted sections were stored at -80˚C. RNA sequencing data from human DRG was reanalyzed from a previous study (77, 78).

Immunohistochemical staining for CGRP in human DRG and spinal cord

For immunohistochemistry, 6 μm sections were deparaffinized and hydrated through graded alcohols to distilled water, followed by antigen retrieval using pH 6 citrate buffer at 70°C for 40 minutes. Primary anti-CGRP antibody was from Peninsula Laboratories (T-4239). Sections were then blocked with hydrogen peroxide and a blocking serum and washed in distilled water. Next, the slides were successively incubated with intermediate wash steps using Tris-buffered saline with 0.1% Tween-20. The first incubation was with a rabbit primary antibody (CGRP, Peninsula Laboratories, Catalogue#T-4239), followed by a biotinylated secondary antibody (Rb Ig Vector Laboratories, Newark, CA#BA-1000) and then HRP-conjugated Streptavidin (Strep. HRP Vector Laboratories #SA-5004). Finally, the slides were developed using Fuchsin Red (Dako #K0625; Agilent, Santa Clara, CA) and counterstained with Hematoxylin. All incubations were carried out at room temperature.

In situ hybridization

The RNA-Scope method (Advanced Cell Diagnostics, Newark, CA) was used for *in situ* hybridization analysis of *STX1A*, *TRPV1*, *TAC1* (substance P precursor), and *LIMK1*. RNAScope® Multiplex Fluorescent assay v2 (Advanced Cell Diagnostics) with Tyramide Signal Amplification (Opal™ Reagent Systems; Perkin Elmer, Waltham MA) were used for multiplex *in situ* hybridization as described previously (69). Staining protocols followed manufacturers' recommendations. The following RNAscope® Probes were used. (catalogue numbers as follows: *STX1A*, 543391; *TRPV1*, 415381; *TAC1*, 310711; *LIMK1*, 560051). Image capture from the stained sections was performed with an Axio Imager.Z2 slide scanning fluorescence microscope (Zeiss, Oberkochen, Germany) equipped with a 20X/0.8 Plan-Apochromat (Phase-2) non-immersion objective (Zeiss), a high-resolution ORCA-Flash4.0 sCMOS digital camera (Hamamatsu), a 200W X-Cite 200DC broad band lamp source (Excelitas Technologies, Waltham MA) and 5 customized filter sets (Semrock, Rochester NY) optimized to detect the following fluorophores: DAPI, Opal520, Opal570, Opal620, Opal690. Image tiles (600 x 600 μm viewing area) were individually captured at 0.325 micron/pixel spatial resolution, and the tiles seamlessly stitched into whole specimen images using the ZEN 2 image acquisition and analysis software program (Zeiss). Stitched images were overlaid as individual layers to create multicolored merged composites. To display low magnification images, signal intensity was substantially enhanced for representative images in some cases. This enhancement was performed uniformly throughout the whole section.

***Visualization of RNA-Seq data using heatmaps and scatter* plots**

Hierarchical clustering and heatmap visualizations were performed on expression values (median RPKM, GTEx Analysis v6)(29) for all 7q11.23 WS locus genes in R. Data were scaled in R and clustered according to the ward.D2 method (70) and visualized with the heatmap.2 function.

Transcriptomic analyses of DRG neuronal subtypes expressing Stx1a

Transcriptomic analyses in Figure 2A and 2B were performed using the data from Goswami, et al. 2014 to identify enrichment of *Stx1a* in mouse DRG *Trpv1* lineage and non-lineage neurons (23). Note that there are some developmental differences between *Trpv1* lineage neurons and adult Trpv1+ neurons due to early expression of TRPV1 during differentiation (5, 79). All genes in the WS locus were examined using read counts in the mouse. Additional verification was obtained by analyzing a sorted RNA-Seq dataset also from mouse DRG from Zheng, et al. 2019 (24). The physiologically characterized categories of DRG neurons were described in (71, 72).

Lymphoblastoid cell lines and RNA-Seq.

RNA was extracted from lymphocyte cell lines for 23 children with WS, 40 typically developing children, and 13 children with Dup7 and RNA sequencing was performed at the NIH Intramural Sequencing Center. Stranded Poly-A selected mRNA libraries were constructed from 1 μg total RNA for each sample using the TruSeq Stranded mRNA Kit (Illumina, San Diego, CA) according to manufacturer's instructions except where noted. Amplification was performed using 10 cycles to minimize over-amplified product. Unique dual-indexed barcode adapters were applied to each library. Libraries were pooled in an equimolar ratio for sequencing. The pooled libraries were sequenced on an S4 flow cell on a NovaSeq 6000 using version 1.0 chemistry to achieve a minimum of 49 million 150 base read pairs. The data was processed using RTA version 3.4.4. Alignment of resulting RNASeq fastq files was performed using STAR version 2.6.1 to the GRCh37 genome build. Aligned reads were further processed using QoRTs version 1.3.6 and normalized count values of genes of interest were analyzed using DESeq2 to test for stepwise group differences.

Cell culture

Dorsal root ganglia (DRG) were dissected from all spinal levels of adult male (150–175 g) Sprague-Dawley rats (Envigo, Indianapolis, IN) and the cells were dissociated as previously described (73, 74). Further details are in the Supplemental Methods. Briefly, rats were euthanized by CO_2 asphyxiation and DRGs were collected, trimmed, and then transferred into collagenase solution (1 mg/ml) for incubation at 37°C for 1 hr. The digested DRGs were rinsed with growth medium, centrifuged and dissociated by mechanical agitation. The cells (~30,000/ well) were plated into each well of 12-well culture plates precoated with poly-D-lysine and laminin. Cells were maintained in F-12 media supplemented with 10% horse serum, 2 mM glutamine, 100 μg/ml Normocin, 50 μg/ml penicillin, 50 μg/ml streptomycin, 50 μM 5-fluoro-2′-deoxyuridine, 150 μM uridine, and 30 ng/ml of NGF in 3% CO_2 at 37°C. Growth medium was changed every other day.

Overexpression of Syntaxin 1A

A lentiviral construct containing (1) the CMV promoter, human *STX1A* (NCBI accession BC064644), IRES, and enhanced green fluorescent protein (EGFP); or (2) CMV, IRES, and EGFP were used to enhance STX1A protein expression in neuronal cell cultures, as previously described (74). On day 5 in culture, increasing amounts (1-50ul; ~1 x 10^5 to 5 x 10^6 transducing units) of the lentivirus was added to the growth media. Two days later, the virus was removed, and the cells were grown for an additional 5 days in normal growth media.

Immunoblotting

Tissues or cells were harvested, lysed in RIPA buffer (Santa Cruz Biotechnology; Dallas, TX), sonicated, and cleared of cellular debris by centrifuging at 4000 RPM for 2 minutes. The protein concentration in lysates was quantified using Lowry assay. Protein aliquots were electrophoresed in a 12% SDS-polyacrylamide gel, transferred to a PVDF membrane, Further details are in the Supplemental Methods. and blocked with Tris-buffered saline containing 0.1% Tween-20 (TBST) and 5% nonfat dry milk for 1 h at room temperature under

gentle agitation. Mouse monoclonal antihuman syntaxin 1 antibody (ThermoFisher MA5-17612, 1:1000) and mouse monoclonal anti-vinculin antibody (Millipore Sigma V4505, 1:1000) were added to the blocking solution and incubated for 2 h at room temperature with gentle agitation. Antibody binding was detected following appropriate secondary antibody methods using chemiluminescence. The density of the bands was measured using Quantity One software from Bio-Rad (Hercules, CA) and data were expressed as density of STX1 normalized to vinculin.

Measurement of calcitonin gene-related peptide release

To assess capsaicin-stimulated release from neuronal cultures with varying amounts of STX1A overexpression, the cultures were washed once with HEPES buffer consisting of (in mM) 25 HEPES, 135 NaCl, 3.5 KCl, 2.5 $CaCl_2$, 1 $MgCl_2$, 3.3 D-glucose, and 0.1% bovine serum albumin, pH 7.4 and maintained at 37°C. Cultures were then incubated for successive 10-min intervals with 0.4 ml of HEPES buffer alone (to assess basal release), with buffer containing 30 nM capsaicin (to assess stimulated release), then with buffer alone (to assess return to basal release). After each incubation, the buffer was removed and immunoreactive calcitonin gene-related peptide (CGRP) in each sample was measured by radioimmunoassay using CGRP antibody generated by Michael J. Iadarola (National Institutes of Health, Bethesda, MD)(31) as previously described (74). After the release experiment, the cells in each well were lysed in 0.4 ml of 0.1 M HCl for 20 min and an aliquot was taken to measure total CGRP content in each well. Total content (fmol/well) was calculated by adding the total amount of CGRP released in all incubations to the total amount of CGRP measured from the lysed cells. The release data is calculated as fmol released/well/10 min and expressed as the % of CGRP release.

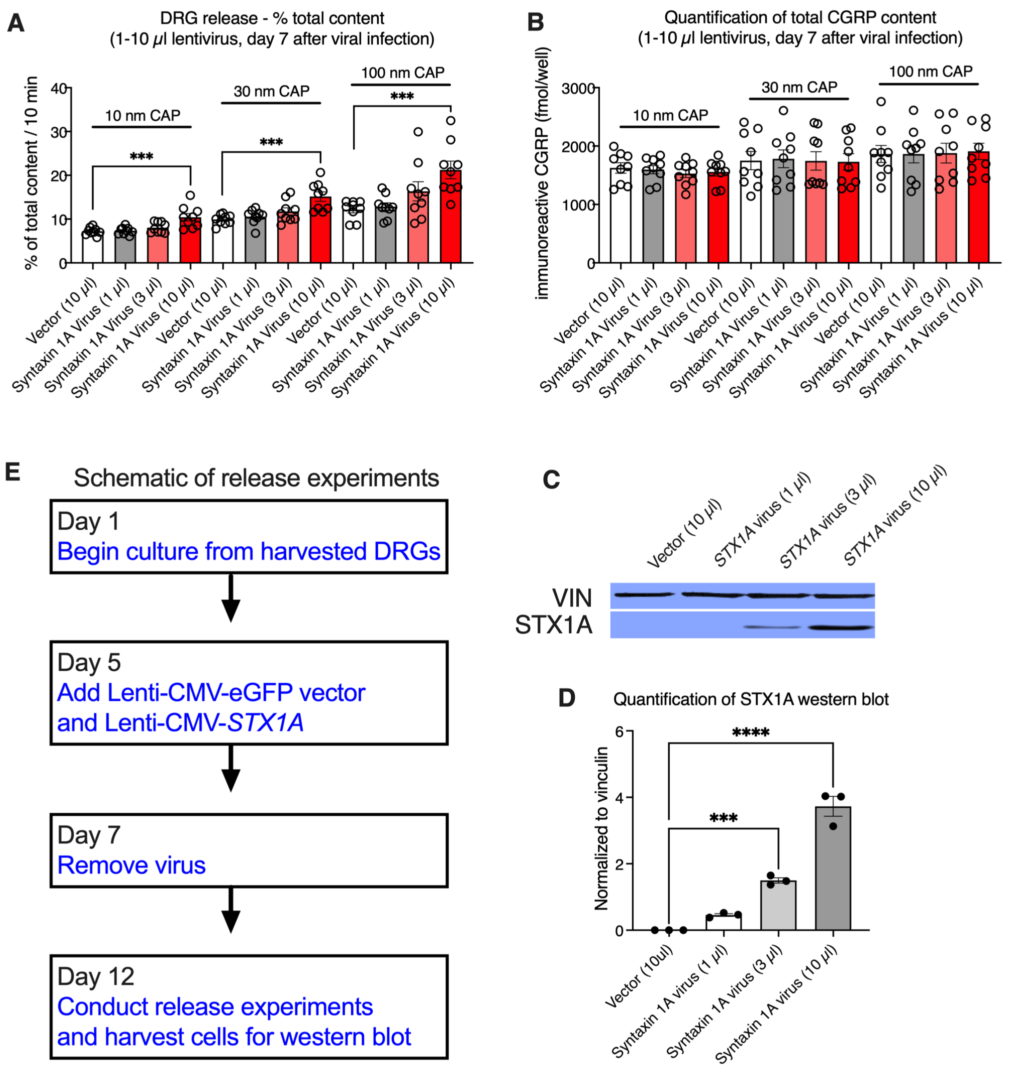

Supplemental Figure 1. Effect of expressing lower amounts of *STX1A* on capsaicin-induced CGRP release from rat primary DGR neuronal culture. (A) CGRP release was evoked with three different concentrations of capsaicin (10, 30 and 100 nmolar) and interacted with three amounts of lentiviral vector. 1 µl of virus = 1 x 10^5 transducing units. Vector expressing eGFP alone was used as the control. (A) For each dose of capsaicin, one-way ANOVA was performed followed by Dunnett's post hoc testing for each condition vs. the vector control. Significant enhancement of release was observed with 10 µl virus at each of the 3 concentrations of capsaicin. (B) Exposure to virus did not affect the primary rat DRG cultures. No alteration of CGRP in the cells on the culture plates occurred. Loss of cells would have been manifested as a decrease in total content. (C,D) Western blot analysis STX1A content. (C) Photograph of the western blot. Note that the basal amount of STX1A protein in the cultures is low. (D) Ascending amounts of vector produce significant, progressive increases in STX1A immunoreactive protein. Higher amounts of vector inhibit CGRP release (see

Figure 5). (E) Schematic of culture time course, viral exposure and capsaicin-induced release.

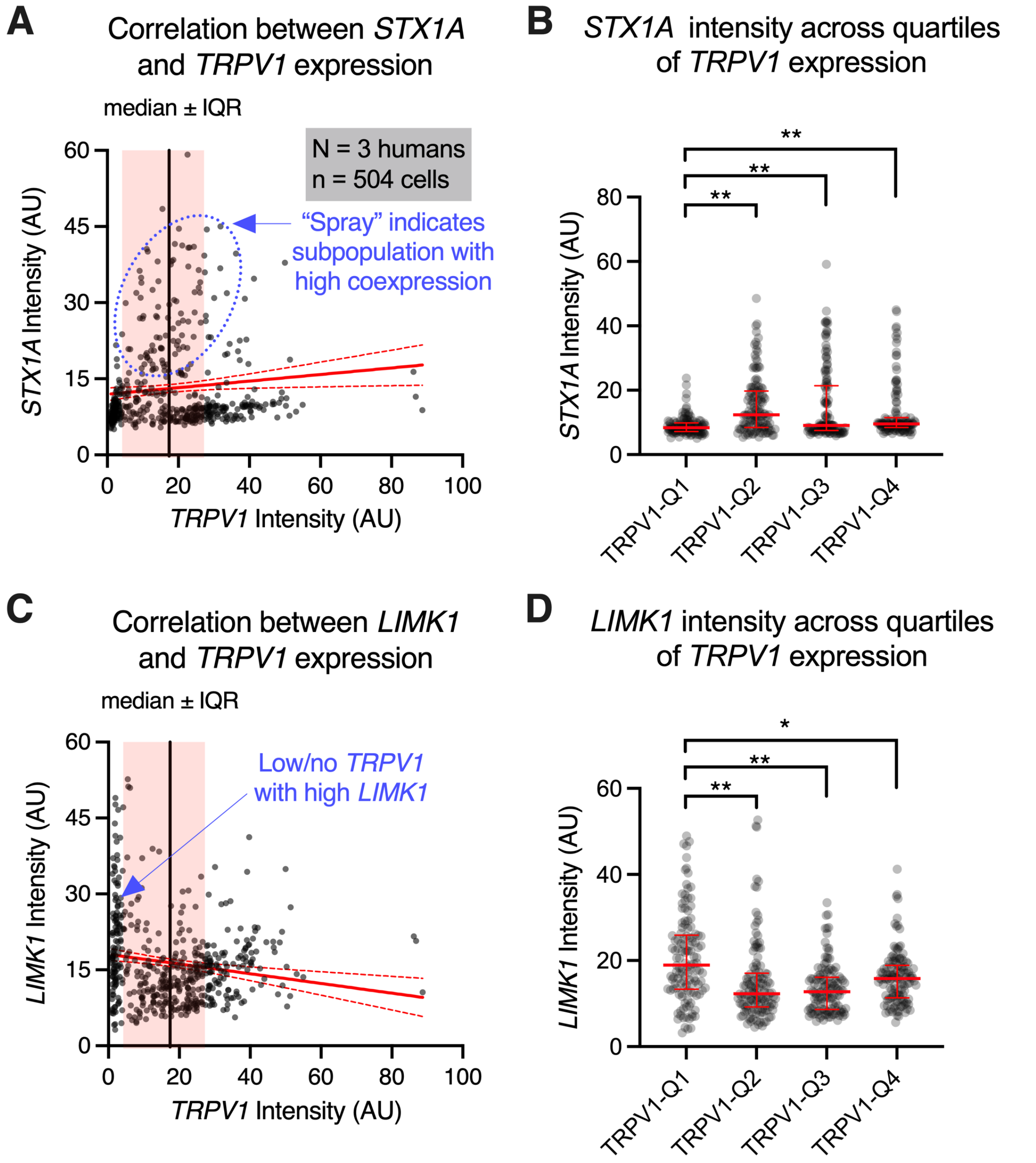

Supplemental Figure 2. Signal intensity and colocalization of *STX1A* and *LIMK1* with *TRPV1* in the human DRG. Binary (yes/no) cell counting showed that *LIMK1* was ubiquitous in the human DRG. However, variation in level of expression of this gene was observed. **A, C.** To quantify the relationships between *STX1A*, *LIMK1* and *TRPV1* expression, correlation plots were generated. These plots suggested a subpopulation of highly expressing *TRPV1*+ neurons that also expressed high *STX1A* (blue oval in panel A). Cells were divided into quartiles of *TRPV1* expression by measuring puncta intensity in ImageJ. This analysis suggests that the cells expressing the least *TRPV1* have

less *STX1A* than other cell populations, indicating that there may be subpopulations in which these two genes are correlated. **C, D.** Conversely, *LIMK1* appeared to be highest in cells with low or no *TRPV1*. **B, D.** Kruskal-Wallis test, Prism Graphpad 9; *, $p < 0.05$; **, $p < 0.01$.
