# Supplementary material for: Syntaxin1A overexpression and pain insensitivity in individuals with 7q11.23 duplication syndrome
Source: JCI Insight. 2024 Feb 22;9(4):e176147. doi: 10.1172/jci.insight.176147 (PMC10967379; doi:10.1172/jci.insight.176147)
Supplement: Unedited blot and gel images [file jciinsight-9-176147-s217.pptx]

## Slide 1
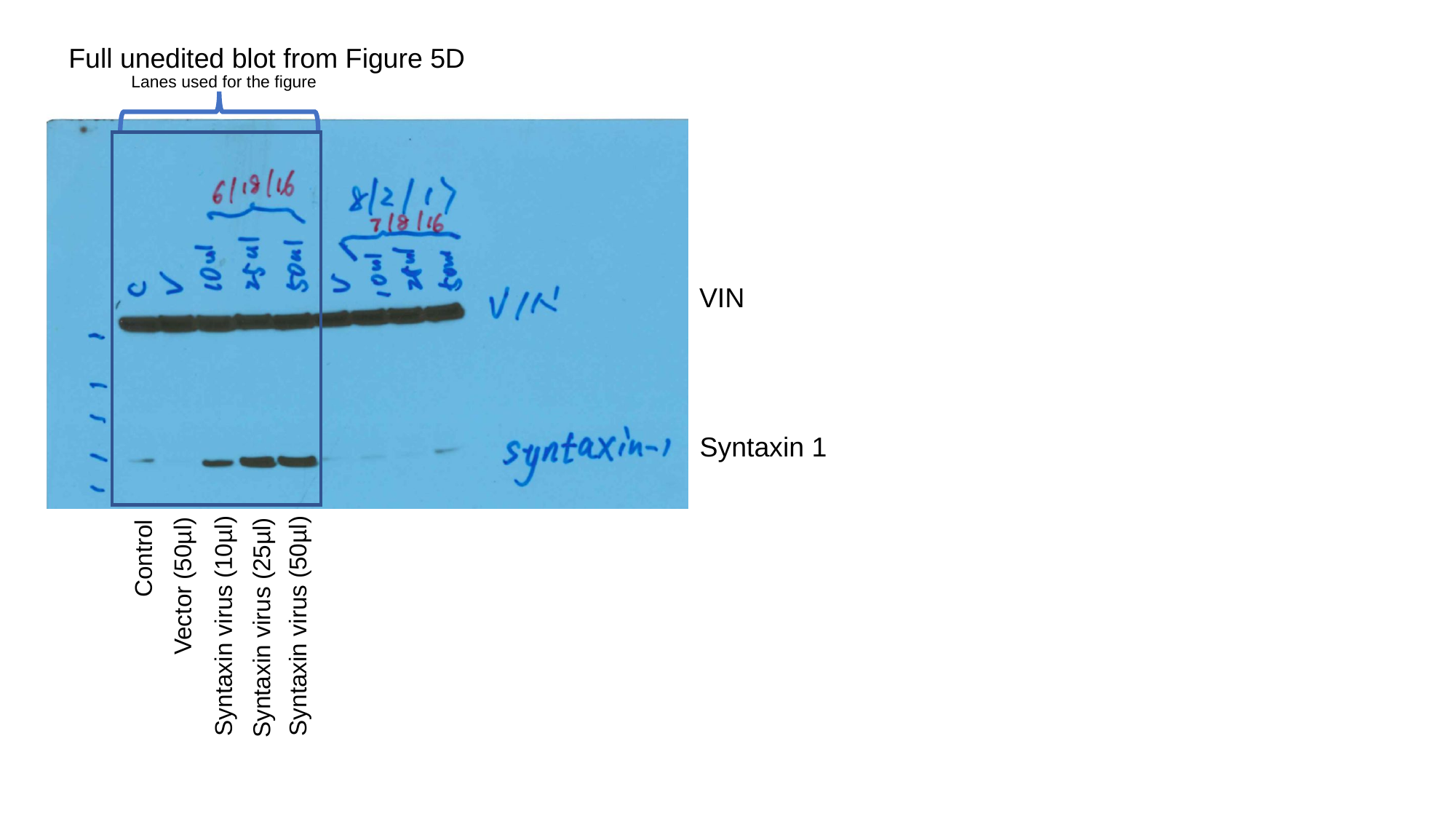

Full unedited blot from Figure 5D
Lanes used for the figure
VIN
Syntaxin 1
Control
Vector (50µl)
Syntaxin virus (50µl)
Syntaxin virus (10µl)
Syntaxin virus (25µl)

## Slide 2
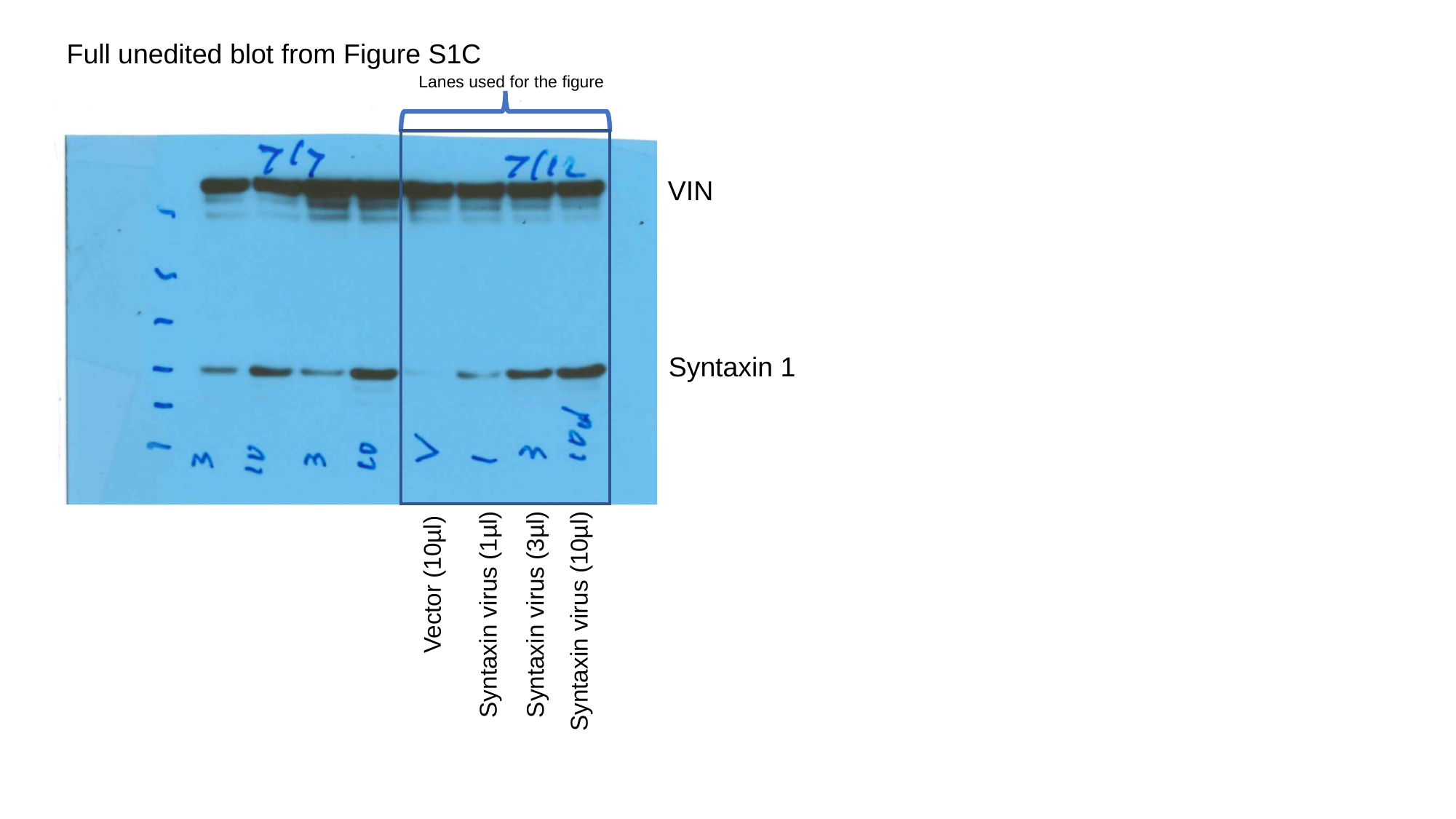

Full unedited blot from Figure S1C
Lanes used for the figure
VIN
Syntaxin 1
Vector (10µl)
Syntaxin virus (1µl)
Syntaxin virus (10µl)
Syntaxin virus (3µl)
